# Fast Multi-Focus Fusion Based on Deep Learning for Early-Stage Embryo Image Enhancement

**DOI:** 10.3390/s21030863

**Published:** 2021-01-28

**Authors:** Vidas Raudonis, Agne Paulauskaite-Taraseviciene, Kristina Sutiene

**Affiliations:** 1 Department of Automation, Kaunas University of Technology, Studentu 48, 51367 Kaunas, Lithuania; vidas.raudonis@ktu.lt; 2 Department of Applied Informatics, Kaunas University of Technology, Studentu 50, 51368 Kaunas, Lithuania; 3 Department of Mathematical Modelling, Kaunas University of Technology, Studentu 50, 51368 Kaunas, Lithuania; kristina.sutiene@ktu.lt

**Keywords:** image fusion, multi-focus, embryo development, data reduction, deep learning, convolutional neural networks, laplacian pyramid, correlation coefficient maximization

## Abstract

Background: Cell detection and counting is of essential importance in evaluating the quality of early-stage embryo. Full automation of this process remains a challenging task due to different cell size, shape, the presence of incomplete cell boundaries, partially or fully overlapping cells. Moreover, the algorithm to be developed should process a large number of image data of different quality in a reasonable amount of time. Methods: Multi-focus image fusion approach based on deep learning U-Net architecture is proposed in the paper, which allows reducing the amount of data up to 7 times without losing spectral information required for embryo enhancement in the microscopic image. Results: The experiment includes the visual and quantitative analysis by estimating the image similarity metrics and processing times, which is compared to the results achieved by two wellknown techniques—Inverse Laplacian Pyramid Transform and Enhanced Correlation Coefficient Maximization. Conclusion: Comparatively, the image fusion time is substantially improved for different image resolutions, whilst ensuring the high quality of the fused image.

## 1. Introduction

The tracking of live cell activity is getting more and more important task for the investigation of cell biology. The information about cell activity gives us a valuable understanding of dynamic cellular phenomena. One of the most challenging tasks-in vitro fertilization (IVF). The success of IVF procedures is closely linked to many biological and technical issues. The fertilisation and in vitro culturing of embryos are dependent upon an environment that should be stable and correct with respect to temperature, air quality, light, media pH and osmolality. At the end of this procedure, there are several embryos, which consequently leads to a problem of choosing the best embryo that is likely to give the greatest success of pregnancy and should be transferred to the uterus. Typically, a healthy embryo progresses consistently through known development stages, has a low percentage of cell fragmentation, neither multinucleation nor asymmetric or reverse cleavage are observed. Embryo selection on Day 2 or 3 is usually based on morphological appearance, assessing the size of cells in blastomere, morphokinetics and the degree of fragmentation. For instance, if the number of cells is four, the fragmentation percent is less than 10 percent and the cells are symmetrical, then the quality of embryo is considered as the best one. Transferring embryos at the blastocyst stage (from Day 5 to 6) has a high potential for pregnancy, whilst keeping a low probability of multiple pregnancy [1]. However, to make a decision manually about the quality of embryo from its visual information is not an easy task. Therefore, the computer-assisted algorithms could be developed for automated embryo quality assessment by taking different factors into account.

**Figure 1:**
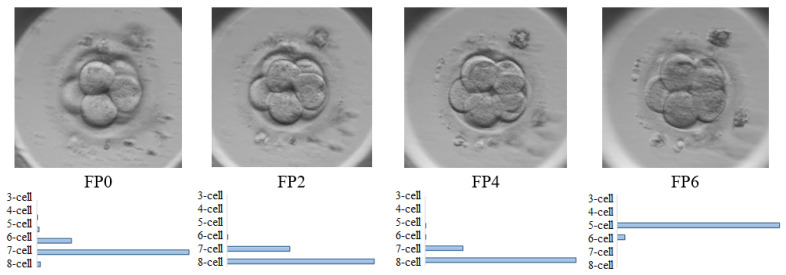
Examples of 8-cell embryo captured in FP0, FP2, FP4 and FP6 focal planes.

The information about cell activity gives us a valuable understanding of dynamic cellular phenomena but the automatic tracking of cells remains a challenging problem in computer vision due to several factors, such as the topological changes of cells (rapid cells deformation), artifacts, noise or blurred parts in the image. Recently deep learning approaches have been recognised as an impressive tool for solving various biomedical image processing problems [2,3]. Embryo assessment based on convolutional neural networks (CNNs) allows to achieving high accuracy results >0.98 classifying them into two classes: good- and poor-quality embryos [4,5]. However, even CNN based approaches encounter difficulties for a multi-class prediction problem, since the embryo quality assessment in a >2-cell stage is still challenging [6,7,8]. Reasons for this may be due to the noise in the image, highly overlapped cells or poor quality of the image. Image processing algorithms detect the embryo based on its size, relative darkness compared to the surrounding area (edge detection), and the diversity of its texture, therefore the results strongly depend on the embryo images. Moreover, often solutions are made based on a single image, however it has been noticed that all cells may not be accurately visible in a single image. One of the reasons is that some cells are focused at a particular distance from the camera, whereas others may be defocused and blurred. This may lead to classification errors, especially when we have 3 and more cells in the image. The cleavage of cells may appear in different 3D directions during embryo development, and different focal planes (FP) highlight different details of embryo. The sharp edges of individual embryo cell usually are not clearly visible in all focal planes. For example, Figure 1 demonstrates that in 8-cell embryo stage only five cells are clearly visible in FP6 focal plane. Therefore, to identify the exact number of cells, each image from different focal plane must be analysed separately. Approximately more than 120 thousand of images are generated, which means that the analysis of such amount of data may require more than one day for one embryologist, given that each image has to be individually evaluated.

One of the possible solutions is to capture a sequence of images focused at different positions and fuse them into a single all-in-focus image. As such, the most direct approach to solve this problem is to use a multi-focus image fusion. By employing the image fusion technique, it is possible to combine two or more images which have some defocused and blurred regions in order to create an all-in-focused image.

To automate this process, we contribute to this field by proposing a fast and effective data reduction approach that is based on the fusion of multi-focus images. The fusion of the images involves the usage of deep artificial neural network that ensures data reduction up to seven times and fast fusion time. The data reduction is done by fusing each individual focal plane images in one highly detailed image, which keeps all-important details from each focal plane. The focal fusing is acquired using U-Net (autoencoder) architecture of deep neural networks. The empirical evidence shows that the proposed approach, being comparatively fast, is able to preserve the information relevant to cell detection, even the data amount is reduced up to seven times.

## 2. Related Works

Recently, many techniques have been developed in the field of image processing [9]. Particularly, all these approaches can be classified into three groups: spatial domain (based on pixel value), transform domain (based on frequency components), and deep learning methods. Spatial domain image fusion methods select pixels or regions from the focused parts in the spatial domain to compose the final image [10,11]. Different transform domain methods are used for image resolution enhancement through the transformation of the source image into different scales, which then are composed into one fused image [12]. Commonly used techniques are wavelet, curvelet and contourlet transforms, neighbour distance, Laplacian pyramid or gradient pyramid [13,14]. Deep learning methods (specifically CNN based approaches) are often incorporated to solve blurring-effect problems through the ability to learn the focus measure to recognize the focused and defocused pixels or regions in source images [15,16]; to learn the fusion operation to fuse a pair without the need for ground truth fused images [17,18]; to learn the direct mapping between the high-frequency and low-frequency images of the source and fusion images [19], and so forth. All these methods can be used to obtain the best focus image from a set of captured microscopic images, but the performance time is the essential factor for the embryo selection task. Speed of microscopic image processing by focus stacking is highlighted by many researchers [11,20], but not less relevant are image spectral information, features to be retrieved or similarity based metrics. For our problem, the latter metrics are important as far as it can affect the correct embryo identification regarding the number of cells. Existing algorithms have proven to be effective in obtaining the best-in-focus image; however, the processing time highly depends on the image objects, visual and color complexity of the image. Moreover, even super-fast methods demonstrate their superiority within specific problem domain. For example, two images of 460 × 610 resolution can be fused in 0.16 s (256 × 256 images per 0.03 s), but the approach is applicable only for merging pairs of different sources-visible and infrared images with different contrast, brightness and sharpness level [21], and usually fails to determine the perfect boundaries for multiple images. Moreover, the review of published papers revealed that most investigations have been performed using images of single object or a few well-separated objects. For example, ρ-CNN approach resulted in average processing time of 7.5 s on four pairs of 640 × 480 image fusion [15]; a region-based algorithm can process seven images of 640 × 480 resolution in 2 s [22]. The performance time estimated for 10 images of 320 × 240 resolution is 4.6 s using a robust sparse representation model with a Laplacian regularization, but the time jumps up to 31.6 s if the stock is increased to 20 images [23]. For microscopic images, some techniques allow reducing the processing time to 1.35 s (using seven layers Laplacian pyramid for 720 × 480 images) [13].

## 3. Multi-Focus Image Fusion Framework

The detailed explanation on multi-focus acquisition hardware is given in this section. Then, the fusion method and the architecture of the built U-Net deep neural network are presented. Two alternatives methods, that is, Inverse Laplacian Pyramid Transform and Enhanced Correlation Coefficient Maximization, which are used to compare with the proposed approach, are described as well. The section ends with a brief description of quantitative metrics used in this study to determine the similarity between a pair of images.

### 3.1. Hardware Setup for the Acquisition of Multi-Focus Images

**Figure 2:**
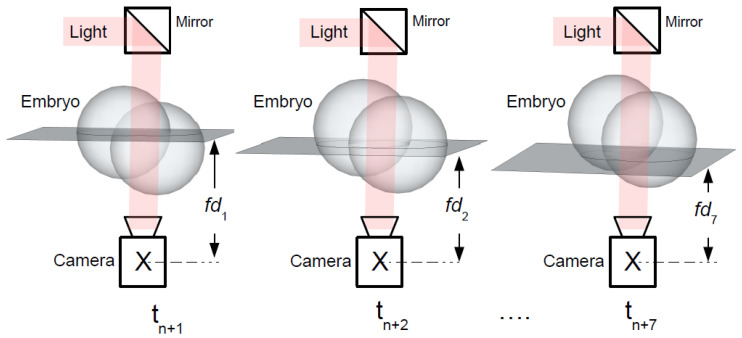
Image capturing process.

The images captured with ESCO MIRI Time-Lapse (TL) incubator are used in this research. TL incubator is a multi-room incubator with a built-in camera and microscope. This incubator provides high quality time-lapse images of embryo development in a real-time without a need for external manual inspection under microscope. Ongoing monitoring of early-stage embryo provides detailed morphokinetic data throughout embryo development, which is not available on routine spot microscopic evaluation. TL incubator has a monochrome 8bit video camera with the resolution of 1280 × 1024 pixels. Built-in microscope has magnification of 20× times, using Zeiss optics. The image recordings of embryo development is executed up to five days with five min intervals in seven different focal planes. The functional scheme of image acquisition process is shown in the Figure 2.

Modern time-lapse incubators, such as ESCO Miri TL, have optical microscopes that capture images of a human embryo at seven different focal planes. The images of each focal plane are taken as fast as possible ($t_{n+1}$ ≤ $t_{n+2}$ ≤ ... $\leq t_{n+7}$) at the different focal distance, that is, from $fd_{1}$ to $fd_{7}$. Each set of focal planes represents 3D object (early-stage embryo) with 7 images. Therefore, each image taken at different focal plane has different information and details about early-stage embryo development at different stages of growth (see Figure 2). Embryologists must evaluate each individual image in the sequence and decide which embryo has abnormal behavior. It is a complicated task because not only the embryo can behave in an unpredicted manner, but also because of the massive image data set which is very difficult to analyse manually. Fully loaded Esco Miri TL6 incubator can generate up to 120 thousand of images per one IVF cycle (84 (number of individual embryos) × 120 (h) × 60 (min)/5 (minutes interval)).

### 3.2. Data Preparation

The experimental study has been carried out using 4000 sets of images that represent 200 individual embryos in different stages, ranging from Zygota up to 8-cell stage. The entire image data set has been partitioned randomly into two subsets, with 80% for training and 20% for test. One data set consists of seven images taken at seven different focal planes and one focus-stacked image, which was generated using Laplacian pyramids [14].

The focus-stacked images were used as target vector in this research study. The data set was prepared by skilled embryologists. All images in the data set were carefully examined and labeled. All irrelevant data (images of very low resolution, images without embryo, or images with occluded embryo with a material that does not belong to embryo) were excluded. The training and testing data sets were formed by randomly selecting sample (images) of each cell stage. These images were fed into the proposed model, which has been trained and tested using NVidia GeForce GTX 1080 GPU processor and implemented in an open-source machine learning TensorFlow framework (version 1.15).

### 3.3. Multi-Focus Image Fusion Approach Using U-Net Architecture

**Figure 3:**
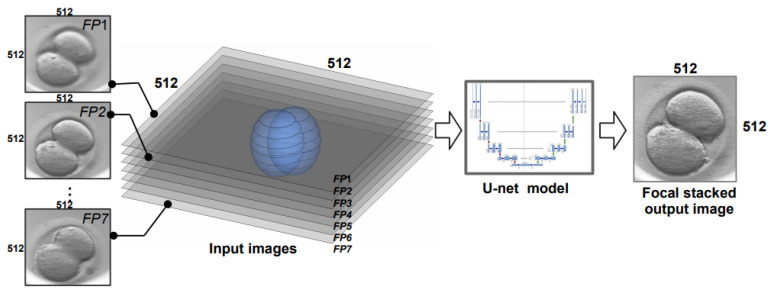
Functional diagram of multi-focal image fusion method.

The basic concept behind the proposed method is the use of autoencoder, such as U-Net, which takes seven focal planes as an input signal and generates one image as an output $I_{U}$. The output image is a result of focal stacking that preserves all import details for each focal plane (see Figure 3).

**Figure 4:**
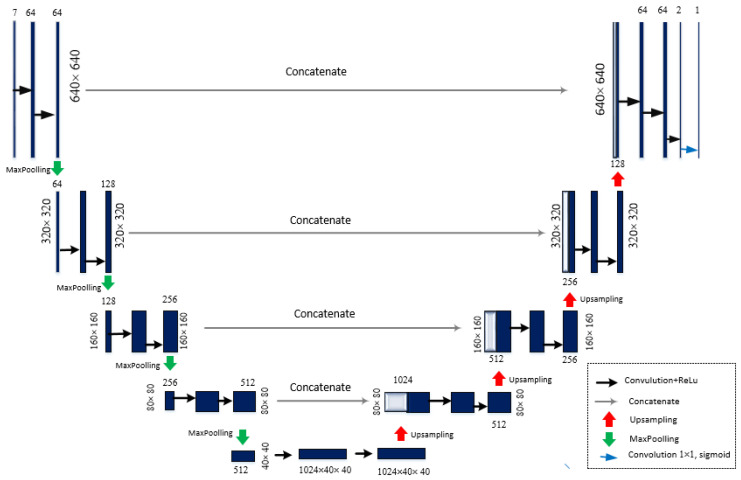
Structure of U-Net architecture (in case of 640 × 640 image).

U-Net convolution network was developed for biomedical image segmentation [24]. This model is based on encoder-decoder framework with skip connections included. In general, the architecture consists of the four phases: convolution with max-pooling, upsampling, concatenation, and again convolution. U-Net structure is organized in two paths: the down-sampling path (encoder) and the up-sampling (decoder) (see Figure 4). Downsampling path captures the contextual information, while up-sampling recovers the spatial information. The input layer in down-sampling path receives seven images of embryo, therefore it has a shape of 640 × 640 × 7 (in case of 640 × 640 image). Input layer is then followed by two 3 × 3 convolution ReLu layers. The next layer is max-pooling of 2 × 2 window size and a stride value of 2.

Down-sampling basically means converting a high resolution image to a low resolution image. In the experiments, the images of four different resolutions have been tested: 480 × 480, 512 × 512, 640 × 640, and 720 × 720. Therefore, the image before pooling, for example, the size of 640 × 640 × 64, is mapped into 320 × 320 × 128 image after max-pooling is applied. The size of the image gradually reduces, while the depth gradually increases, until the image size is 40 × 40 × 1024. Finally, so called “bottleneck” is reached in a fifth layer, which is between the down-sampling and up-sampling paths. It is built from two convolutional layers with 0.5 probability dropout layer.

In up-sampling (right-hand side) path, a low resolution image is converted to a high resolution image. A skip connection is used to transfer local information by concatenating feature maps from the down-sampling path with feature maps from the up-sampling path. After every concatenation two consecutive regular convolution layers are inserted. This group of layers is repeated starting from group six to group nine. The last layer is a convolution layer with one filter of size 1 × 1 and the result is a fused 640 × 640 × 1 image $I_{U}$. In total, 29 layers are involved in this architecture including 20 convolutional + ReLU layers, 4 max-pooling layers, 4 up-convolutional layers, and 1 output layer. This model was compiled with Adam optimizer, with the loss function defined as mean squared error.

### 3.4. Alternative Image Fusion Approaches

In this section, two alternatives to the proposed approach are briefly presented: (1) inverse Laplacian Pyramid transform and (2) Enhanced Correlation Coefficient maximization, which both could be used to implement image fusion.

#### 3.4.1. Inverse Laplacian Pyramid Transform

The basic principle of Laplacian pyramids (LP) based approach is the decomposition of the raw image into regions (or sub-images) with different spatial resolution [14]. The fused image is constructed from the sub-images with higher spatial resolution. The Laplacian pyramid is computed from the Gaussian pyramid that is a multi-scale representation of image, which is recursively filtered using different low-pass filters. Gaussian pyramids consist of filtered down sampled images, constructed using separable 1D kernel *h* and a down-sampling factor of 2 (in each direction). Over-complete decomposition is based on difference of low-pass filters, and the image is recursively decomposed into low-pass and high-pass bands. Each level of LP is the difference between two adjacent low-pass images of the Gaussian pyramid [$I_{0}$, $I_{1}$, ..., $I_{N}$ ], that is,

(1) $$b_{k}=I_{k}-EI_{k+1};$$

where $b_{k}$ is *k* level of Laplacian pyramid and $EI_{k+1}$ is an up-sampled smoothed version of $I_{k+1}$ (so that it will have the same dimension as $I_{k}$).

**Figure 5:**
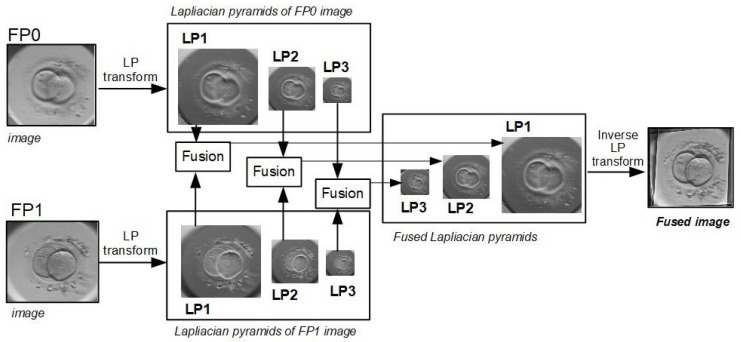
The framework of fusion method based on Laplacian Pyramid transform.

An important property of the Laplacian pyramid is that pyramids completely describe the original image. Laplacian pyramids represent the image edges at every level; so by comparing the corresponding pyramid levels of two images, it is possible to generate the fused image, which merge their respective high-contrast details and keep the fused image as rich as possible. The basic framework of image fusion based on Laplacian pyramid transform is shown in Figure 5.

Figure 5 illustrates fusion of two images only, but the same approach can be applied for seven images of each focal plane. The fusion of FP0 and FP1 images is done in three main steps: (1) Laplacian transformation is applied to each input image, (2) corresponding pyramid levels of FP0 and FP1 images are fused together, and Laplacian pyramid of fused image is acquired, (3) the fused image is reconstructed based on Laplacian pyramid inverse transformation.

#### 3.4.2. Enhanced Correlation Coefficient Maximization

Enhanced Correlation Coefficient (ECC) maximization algorithm is a gradient-based image registration algorithm. This algorithm achieves high sub-pixel accuracy, because of gradient information. ECC algorithm is invariant to a global illumination variation, because it is based on zero-mean normalized cross correlation (ZNCC). Basically, ZNCC is a similarity value between two images $I_{1}$ and $I_{2}$ and is given by

(2) $$ZNCC=\frac{1/NM\left[\sum_{i=1}^{N}\sum_{j=1}^{M}\left(I_{1}\left(i,j\right)-\mu_{1}\right)\sum_{i=1}^{N}\sum_{j=1}^{N}\left(I_{2}\left(i,j\right)-\mu_{2}\right)\right]}{\sigma_{1}\sigma_{2}};$$

where N is the number of rows in the image, M is the number columns in the image. For k=1,2, the average value $\mu_{k}$ and the standard deviation $\sigma_{k}$ of the image are given by

(3) $$\mu_{k}=\frac{1}{NM}\sum_{i=1}^{N}\sum_{j=1}^{M}I_{k}\left(i,j\right)$$

and

(4) $$\sigma_{k}=\sqrt{\frac{1}{NM}\sum_{i=1}^{N}\sum_{j=1}^{M}{\left(I_{k}\left(i,j\right)-\mu_{k}\right)}^{2}}.$$

ECC based fusion of two images is executed throughout two main steps: (1) the input images are aligned based on 2D geometric transformation, which is estimated based on ECC algorithm, (2) the aligned images are fused based on the average values of the corresponding pixels.

### 3.5. Image Similarity Metrics

Image similarity metrics are developed to quantitatively assess the similarity of images, but there is no universal measure which could be used in all applications [25]. Therefore, some of widely accepted quantitative metrics are used in the study to measure the similarity between two images. In case of application, the similarity metrics were computed between the images generated by the proposed focus-stacking algorithm and the test images which were not used for training. Specifically, both test images and training images were generated using LP approach. Suppose $I_{X}$ and $I_{Y}$ denote a pair of these images in general. Then, the image similarity metrics are briefly presented as follows.

- Root Mean Squared Error (RMSE) is commonly used to estimate the difference between two images by directly computing the variation in pixel values. The smaller value of RMSE represents better similarity [26]. Its value is defined as
  (5) $$\mathrm{RMSE}=\sqrt{\frac{1}{MN}\sum_{i=1}^{M}\sum_{j=1}^{N}(I_{X}\left(i,j\right)-I_{Y}{\left(i,j\right)}^{2}};$$
  where *M* and *N* is the width and the height of image.
- Spectral Angle Mapper (SAM) determines the spectral similarity between two spectra by calculating the angle between the spectra and treating them as vectors in a space with dimensionality equal to the number of bands. Small angles between the two spectrums indicate high similarity, where the ideal value of zero indicates the best spectral quality [27]. It is calculated using the following formula
  (6) $$\mathrm{SAM}=\mathrm{acos}\left(\frac{\sum_{i=1}^{L}u_{i}v_{i}}{\sqrt{\sum_{i=1}^{L}u_{i}^{2}}\sqrt{\sum_{i=1}^{L}v_{i}^{2}}}\right);$$
  where *L* is the number of bands, *u* and *v* are two adjacent spectra.
- Peak Signal-to-Noise Ratio (PSNR) is calculated based on RMSE, taking into account maximum possible pixel value of the image. For 8-bit representation, acceptable values for wireless transmission quality loss are considered to be around 20 dB to 25 dB, while in a lossy image range between 30 and 50 dB, where higher is better [28]. The value of PSNR is obtained using
  (7) $$\mathrm{PSNR}=20\;\log_{10}\frac{\max}{\mathrm{RMSE}};$$
  where max is the maximum possible value a pixel can take. The value of max=255 is set when the pixels are represented using 8 bits.
- Universal Quality Index (UQI) represents brightness distortion, contrast distortion and correlation difference between two images. The best value is 1 if the images are equal [29]. The mathematical form of UQI is
  (8) $$\mathrm{UQI}=\frac{4\sigma_{I_{X}I_{Y}}\mu_{I_{X}}\mu_{I_{Y}}}{\left(\sigma_{I_{X}}^{2}+\sigma_{I_{Y}}^{2}\right)\left(\mu_{I_{X}}^{2}+\mu_{I_{Y}}^{2}\right)};$$
  where $\mu_{I}$ is the mean value of the image, $\sigma_{I}$ is the standard deviation the image, and $\sigma_{I_{X}I_{Y}}$ is the covariance between two images $I_{X}$ and $I_{Y}$.
- Structural Similarity Index Method (SSIM) determines the local patterns of pixel intensities between two images taking into account three estimates of luminance, contrast, and structure [30]. The value ranges between −1 and 1, where the ideal value is 1. The value of SSIM is given by
  (9) $$\mathrm{SSIM}=\frac{\left(2\mu_{I_{X}}\mu_{I_{Y}}+C_{1}\right)\left(2\sigma_{I_{X}I_{Y}}+C_{2}\right)}{\left(\mu_{I_{X}}^{2}+\mu_{I_{Y}}^{2}+C_{1}\right)\left(\sigma_{I_{X}}^{2}+\sigma_{I_{Y}}^{2}+C_{2}\right)};$$
  where $\mu_{I}$ is the mean value of the image, $\sigma_{I}$ is the standard deviation of image, $\sigma_{I_{X}I_{Y}}$ is the covariance between two images, while $C_{1}$ and $C_{2}$ are constants. In this study, the values $C_{1}=0.01$ and $C_{2}=0.03$ have been chosen by default.
- Multi-Scale Structural Similarity Index Method (MS-SSIM), which is more advanced form of SSIM, determines the quality based in the terms of image luminance, contrast and structure at multiple scales. The ideal value is 1. The computations are typically performed in a sliding *N* × *N* (by default 11 × 11) Gaussian-weighted window [31].

## 4. Experimental Results

**Figure 6:**
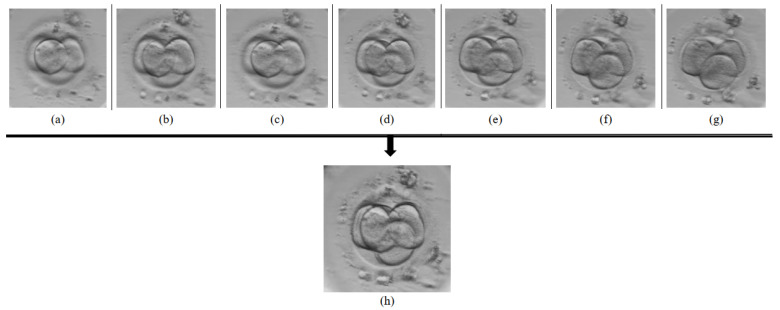
A fused 4-cell embryo image (**h**) generated from seven focal stack images (**a**–**g**).

The proposed multi-focus image fusion approach using U-Net architecture has been tested using seven focal stack images of embryo captured by adjusting the camera at different positions. In Figure 6, seven images of 4-cell embryo are depicted for the demonstration purposes. It can be observed that all seven images capture different focus, consequently the number of visible cells varies from three to four (see Figure 6a–g). Accordingly, the output image $I_{U}$ combines the relevant information from all images into a single fused image $I_{U}$ (Figure 6h) depicting four cells.

**Figure 7:**
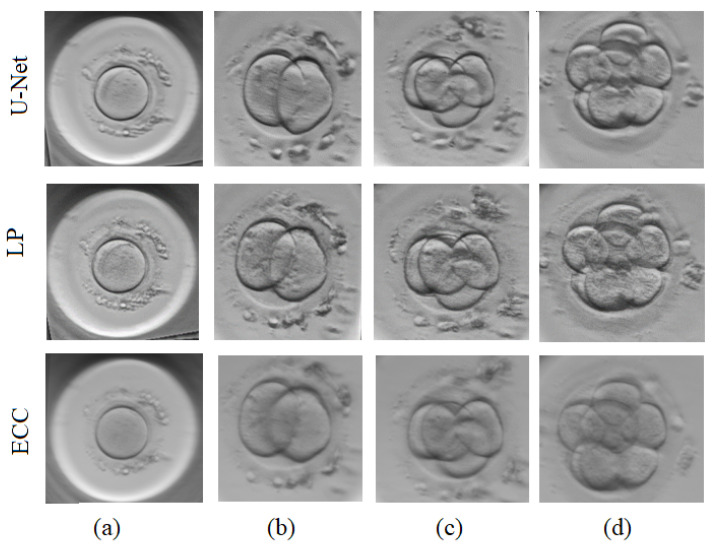
Fused embryo images using U-Net, Laplacian pyramids (LP) and Enhanced Correlation Coefficient (ECC) approaches: (**a**) one cell, (**b**) two cells, (**c**) four cells, (**d**) eight cells.

All three approaches have been tested on the same image pairs including 1-cell, 2-cell, 4-cell and 8-cell embryos (see Figure 7). No obvious difference is seen when looking at the fused images generated by U-Net approach and LP method. Both these methods provide images with sharp edges of cells, clearly visible fragmentation and surrounding artefacts. Comparatively, the fused images generated by ECC differ significantly, with strong blurriness visible relative to the previous set of images.

**Figure 8:**
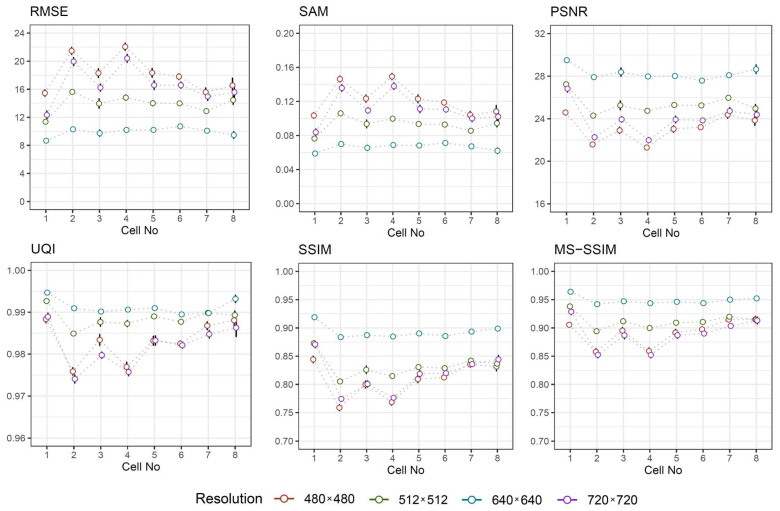
Similarity metrics against cell number for different image resolutions.

To validate the proposed approach, the quantitative metrics (see Section 3.5) are computed to assess the similarity between two images, that is, image $I_{LP}$ generated by inverse Laplacian pyramid transform (see Section 3.4.1) and image $I_{U}$ generated using the proposed approach employing deep learning technique—U-Net convolutional network architecture. Based on these metrics, the higher similarity between images is achieved when (a) RMSE and SAM close to zero; (b) PSNR obtains higher value; (c) UQi, SSIM, and MS-SSIM approaches to one. In the experiment, the mean values of the aforementioned metrics and their confidence intervals for a considered sample are estimated and depicted in Figure 8.

From Figure 8, one can observe that images of higher resolution are quantified with better similarity. In most cases, the metrics are substantially improved when the image resolution is higher, such as 720 × 720 pixels. Another important observation is that the metrics seemed to be invariant to cell number in the image, especially when the image resolution is high. However, for low resolution images the metrics tend to vary for lower number of cells. Furthermore, the estimated confidence intervals for a mean are quite narrow, which implies the precision of a particular estimate, allowing us to be confident about the experimental results.

**Figure 9:**
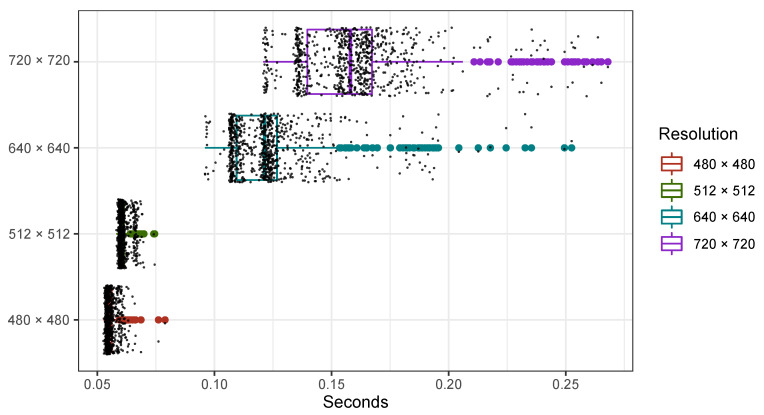
Processing times of the proposed approach for different image resolutions.

Next important question to be addressed in this study—the processing time of the proposed approach. In particular, Figure 9 shows the distribution of processing times for different image resolutions.

As it was expected, higher image resolution requires more processing time (see Figure 9). Comparatively, the time estimated for the highest resolution, on average, is nearly three times larger than the time estimated for the lowest resolution. On the other hand, the mean values of processing times are less than 165 ms, which is really fast in comparison to the published results (see Section 2), especially also having in mind that a deep learning technique has been employed.

## 5. Discussion

The automation of early-stage embryo detection not only requires to be precise but also fast, having in mind that the algorithm should process a large number of image files of different qualities in a reasonable amount of time. Moreover, the embryo detection is complicated by its topological changes during the development. Therefore, in this section we additionally explore the computational time required for image fusion. In particular, we compare the fusion time of the proposed multi-focus image fusion approach using U-Net architecture with alternative approaches presented in Section 3.4. Notably, all three approaches have been fed with the same image set.

**Figure 10:**
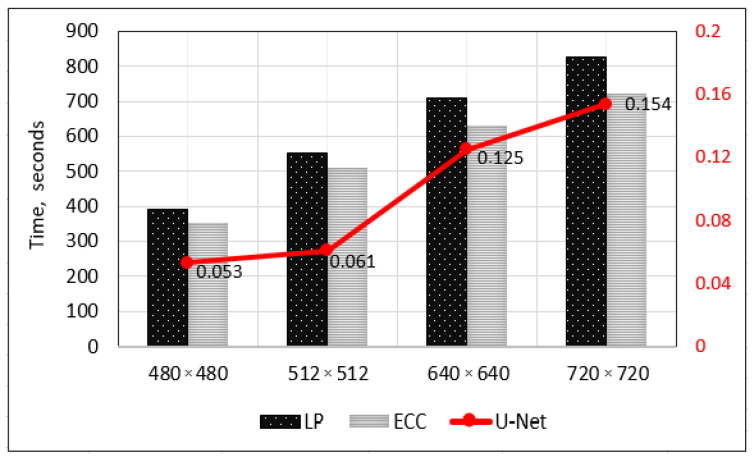
Comparison of fusion times using the proposed multi-focus image fusion approach using U-Net architecture, and its two alternatives such as Laplacian pyramid transform and ECC method for different resolution images.

**Figure 11:**
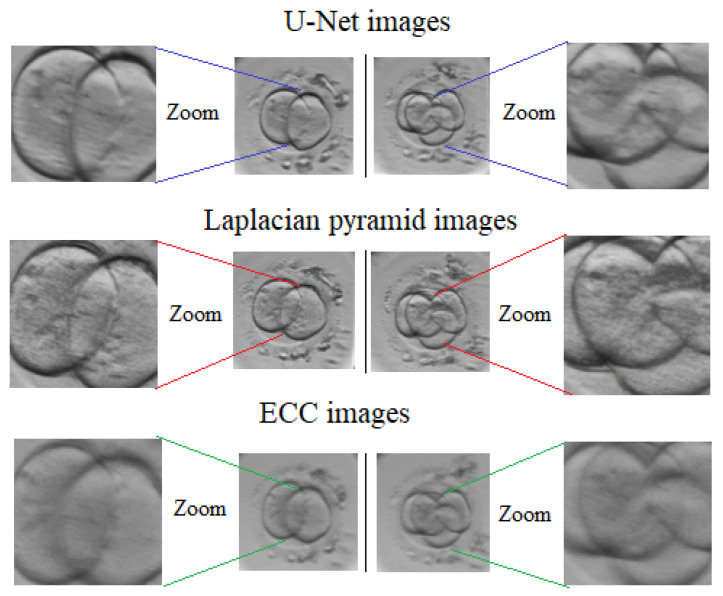
Comparison of the fused images using the proposed multi-focus image fusion approach using U-Net architecture, LP transform and ECC method for 2-cell and 4-cell embryos.

As can be seen from Figure 10, the average running times required for image fusion processing using are comparatively similar for Laplacian pyramid transform and ECC method. However, ECC method performs faster on average 10%. In fact, these observations reveal that the proposed multi-focus image fusion approach using U-Net architecture is super fast since the processing time has been substantially improved despite the use of deep learning technique, which is typically time consuming. For demonstration purposes, Figure 11 visually compares the output from the proposed approach and its two alternatives for 2-cell and 4-cell embryos.

From Figure 11, it can be observed that ECC approach resulted in a blurry image fading out the contours of the cell. Comparatively, the fused images based on Laplacian pyramid transform have the highest contrast and brightness level, while the images obtained using the proposed approach have relatively sharper contours with blurred parts observed in the image, from a subjective evaluation of view point. This is the price we pay for the fast image fusion processing time, which is not so high having in mind that the estimates of similarity metrics (see Section 4) have confirmed the strong similarity, especially for high resolution (720 × 720) images. The narrow application area is the biggest limitation of our fusion method presented in this paper. Notably, the proposed approach is limited by the dedicated training dataset, which consists of entirely early-stage embryo images. Therefore, the presented approach can be applied to other types of images after adaptation only. It should be also noted that the direct comparison of methods mentioned in SOTA is complicated due the fact that already published papers on deep structures of the neural networks include mostly two images as inputs and generate one fused image. We propose U-Net based algorithm, which generates one focus-stacked image from seven images taken at seven different focal planes (one input per image). The major modification of SOTA structures of deep neural networks is needed in order to provide the adequate comparison. Therefore, the method developed in the current paper is compared with those that can work up to seven images.

**Figure 12:**
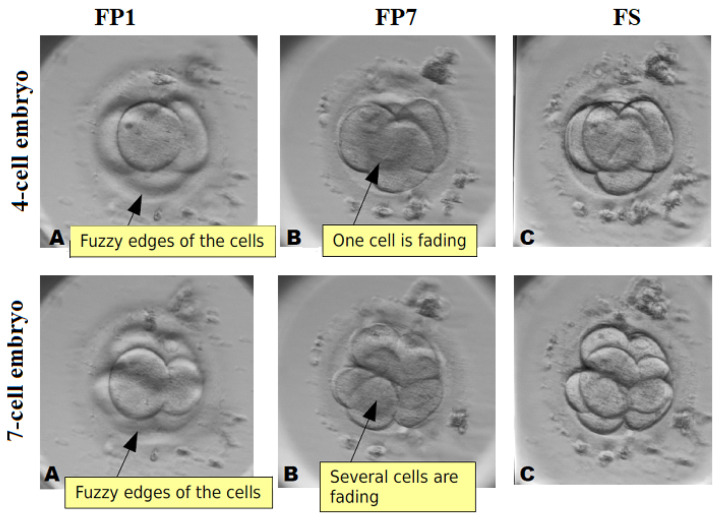
(**A**) early-stage embryo taken at the first focal plane (FP1), (**B**) early-stage embryo taken at the seventh focal plane (FP7), (**C**) fused image using the proposed algorithm (FS).

Additionally, we demonstrate why the images of all seven focal planes are important for early-stage embryo image enhancement. The images of 4-cell embryo and 7-cell embryo are shown in Figure 12. It is clearly visible that cells captured in FP1 image or in FP7 image have fuzzy edges and unclear boundaries Figure 12A,B. The focus-stacking algorithm presented in the paper preserves individual properties from all FP images and presents them in one fused image (see Figure 12C), where all cells are surrounded by clear and sharp boundaries.

The proposed focus-stacking algorithm reconstructs the embryo image correctly without losing any information. The future work will include more detailed investigation of reconstruction accuracy, which involves manual evaluation of fused images by several embryologists. Our primary research with two experienced embryologists has shown that the whole development of single early-stage embryo has been evaluated on average three times faster analyzing the fused image instead of seven focal planes separately.

## 6. Conclusions

The present study has addressed the challenges that are known in order to automate early-stage human embryo detection in time-lapse microscopy image sequences. The complexity comes from the data processing, which is due to cell overlapping, background clutters, inhomogeneous intensities, and image artifacts. Moreover, the algorithm is also challenged by a large number of images, which needs to be processed relatively quickly.

Therefore, we proposed a new approach for data reduction and fast fusion by employing deep neural networks, specifically U-Net architecture (autoencoder). In the study, this approach was verified in different ways. First, the results of this proposed framework were compared with images generated by inverse Laplacian pyramid transform and Enhanced Correlation Coefficient Maximization. Based on the selected similarity metrics, it was concluded that the differences between images tend to diminish to a minimum if a higher resolution image was fed to the algorithm. Secondly, by exploring the similarity metrics against the cell number, it was observed that the proposed approach becomes invariant to the cell number if the image of higher resolution was considered. This implies the consistent performance of this approach despite the certain stage of embryo development. Finally, it was determined that the mean values of image fusion process using the proposed approach for different image resolution are substantially reduced comparing them with times achieved by two alternatives approaches implemented on the same data sample.

## Data Availability

Restrictions apply to the availability of these data. Data were obtained from a private IVF laboratory (ESCO MEDICAL Ltd., company code 303705851, Draugystes str. 19, 51230 Kaunas, Lithuania) and then shared with the research group “Smart Automatic Control Systems” led by prof. Vidas Raudonis for research purposes under Data Use Agreement established on 3 September 2018.

## Abbreviations

The following abbreviations are used in this paper:

CNNs	Convolutional Neural Networks
ECC	Enhanced Correlation Coefficient
IVF	In-Vitro Fertilization
LP	Laplacian pyramid
MS-SSIM	Multi-Scale Structural Similarity Index Method
PSNR	Peak Signal-to-Noise Ratio
SAM	Spectral Angle Mapper
SSIM	Structural Similarity Index Method
TL	Time-Lapse
UQI	Universal Quality Index
